# Unpacking how virtual reality stimulates learning through nursing students’ experiences: a qualitative study

**DOI:** 10.1186/s12912-026-04539-6

**Published:** 2026-03-13

**Authors:** Nai-Hui Chien, Yi-Ya Chang

**Affiliations:** 1 Department of Nursing, Chang Gung University of Science and Technology, Taoyuan City, Republic of China (Taiwan); 2 Department of Nursing and Center for Health and Geriatric Care Research and Development, Chang Gung University of Science and Technology, No.261, Wenhua 1st Rd., Kueishan Dist, Taoyuan, 33303 Republic of China (Taiwan)

**Keywords:** Experience, Learning, Nursing students, Nursing education, Virtual reality, Qualitative study

## Abstract

**Background:**

The popularity and use of virtual reality in nursing education is on the rise. However, how virtual reality assists nursing students’ learning is still unclear. This study aimed to explore how virtual reality stimulates learning through undergraduate nursing students’ experiences.

**Methods:**

A qualitative descriptive study was conducted. A purposive sampling was employed to recruit 36 second-year nursing students at a university in northern Taiwan. A semi-structured interview guide was used. Three focus groups were digitally recorded and transcribed verbatim. Braun and Clarke’s thematic analysis was adopted to analyze the interview content.

**Results:**

Three themes emerged: educational entertainment, triggered motivation for enhanced learning, and technological concerns and challenges.

**Conclusion:**

This study contributes qualitative insights into students’ attitudes and experiences with virtual reality and extends knowledge about how virtual reality helps nursing students’ learning. Virtual reality can be implemented in nursing education to increase nursing students’ learning motivation. Educators and developers could design different virtual reality simulators to teach different nursing skills to nursing students. Future research can focus on developing diverse virtual reality interventions to support student learning across a broader range of nursing competencies and clinical scenarios.

**Clinical trial number:**

Not applicable.

## Introduction

The rapid development of technology has changed teachers’ teaching methods and students’ learning approaches. Teachers have started incorporating various technologies into their curriculum to assist students’ learning. Among these technological innovations, virtual reality (VR) has been increasingly adopted in nursing education, applied in healthcare training for both nursing students and professionals. Research has shown that VR provides an expanded method of teaching and learning, allowing students to practice skills and achieve mastery in a safe environment [1]. VR, acting as a simulation, ensures the safety of trainees, teachers, and patients, enabling more flexible training in various clinical scenarios [2]. VR has also been shown to allow students to learn crucial healthcare skills and enhance health profession education [3, 4]. VR can be used for clinical skills training for nursing students and job training for nurses [5]. The trend of using VR in nursing education is growing, but further research evidence is required to comprehend the impact of VR on students’ learning fully.

In recent years, research on using VR in nursing education or training has shown that VR can enhance nursing students’ knowledge, learning motivation, confidence and learning outcomes [6–11]. Most studies have focused on quantitative research designs, investigating the learning outcomes and confidence of nursing students/nurses in knowledge/nursing skills after using VR and their acceptance/satisfaction/attitude towards VR [7, 12–17], while the underlying mechanisms of how VR stimulates learning remain underexplored. Specifically, there is limited understanding of how and why VR facilitates learning from students’ perspectives. Because these learning processes are subjective and context-dependent, qualitative inquiry is particularly suited to capture the depth and complexity of students’ experiences that quantitative measures cannot fully reveal. Therefore, this study employs qualitative research to explore nursing students’ subjective perceptions and meaning-making processes during VR-based learning, thereby enhancing our understanding of how VR facilitates learning from the learners’ perspectives.

In addition to nursing educators using VR to facilitate teaching and understand nursing students’ learning outcomes, there is a need for more evidence and diverse experiences and perspectives to understand undergraduate nursing students’ experiences and perceptions of using VR for learning nursing skills. Therefore, gaining a deeper understanding of nursing students’ experiences with using VR is crucial to nursing educators for designing appropriate teaching strategies.

## Methods

### Aim

This study aimed to explore how VR stimulates learning through undergraduate nursing students’ experiences.

### Design

A qualitative descriptive design was used for this study. This study was conducted at a university located in northern Taiwan from March to April 2022. The Consolidated Qualitative Research Reporting Criterion (COREQ) guidelines for reporting qualitative research were used in this study.

### Participants

Purposive sampling through posted announcements was utilized to recruit 36 second-year nursing students who had experienced using an intravenous injection virtual reality simulator from a total of 128 eligible students. The inclusion criteria were as follows: (1) students who enrolled in the Nursing Fundamentals and Laboratory course; (2) those who had completed the intravenous injection course; (3) those willing to provide written informed consent. The exclusion criterion was students who did not participate in the VR simulation for intravenous injections.

Prior to the interviews, all participating students had received intravenous injection skills training using the traditional learning method first, followed by the virtual learning method. All participants in this study received proper orientation and instruction on how to use the VR simulator before the actual learning experience. The orientation included a demonstration of the VR equipment and guidance on operating the controllers to help students familiarize themselves with the virtual environment. Teaching assistants were available to assist students while they engaged in the VR experience.

To ensure voluntary participation free from coercion and to avoid potential grade-related pressure, participant recruitment and focus group interviews were scheduled to occur only after the completion of the course and final grade submission. This timing eliminated any perceived influence on academic evaluation. Participation in this study posed no physical, psychological, or grade-related risks to participants. Students’ decisions to participate or decline had no impact on their academic standing or relationship with the instructor.

### Data collection

A total of 36 students participated in this study and were organized into three focus groups, with 12 students in each group, for the focus group interviews. A semi-structured interview guide (Table 1) was developed in consultation with the research team to explore the nursing students’ perspectives on their experiences and concerns regarding learning nursing skills through virtual reality compared to traditional learning methods. The focus group interviews lasted between 60 and 75 min, conducted by the second author who is a female nursing teacher. The second author was an interviewer in focus groups, a nursing faculty member with over 25 years of teaching experience in nursing and expertise in qualitative research for more than 10 years, who taught nursing students to learn intravenous injections by VR and took detailed notes during the interviews. To alleviate academic performance pressure on the nursing students during the focus group interviews, the interviews were scheduled to take place after the completion of the course. At the time of the interviews, the nursing students had already completed their first clinical practicum. All interviews were digitally recorded.

**Table 1:** Semi-structured interview guide

→ What are your experiences of learning nursing skills through virtual reality? → What are the differences that you notice between learning nursing skills through virtual reality and traditional learning? → What did you think about using virtual reality to learn nursing skills? → What did virtual reality learning mean to you? → What expectations or concerns do you have regarding learning nursing skills through virtual reality?	

### Data analysis

All interviews were transcribed verbatim by a research assistant. The second author (Y.Y.C.), who conducted the interviews, verified transcription accuracy by listening to recordings while reading transcripts. The interview transcripts were listened to and read repeatedly to ensure consistency between the audio and the written text and to become familiar with the data before coding. Manual coding was independently performed by the first author (N. H. C.) and the second author (Y. Y. C.). Braun and Clarke’s six-phase thematic analysis approach [18] was adopted to analyze the content of the focus group interviews. This systematic method involves: (1) familiarization with data, which involved repeatedly reading the interview transcripts to gain a comprehensive understanding of the content; (2) generating initial codes, where meaningful segments of data were systematically identified and labeled; (3) searching for themes, during which codes were collated and organized into potential broader themes; (4) reviewing themes, which entailed examining whether the themes accurately reflected the coded data and the entire dataset; (5) defining and naming themes, where the essence of each theme was refined and clearly articulated; and (6) producing the final report, which involved selecting compelling extract examples and relating the analysis back to the research questions and literature.

The evaluative criteria of credibility, dependability, transferability, and confirmability, as proposed by Lincoln and Guba [19], were applied in this study. To establish credibility, three focus group interviews were conducted with nursing students who had experience with an intravenous injection virtual reality simulator. Multiple steps were taken to achieve dependability. The research team reviewed the codes and themes from various perspectives during the data analysis. To resolve discrepancies in coding, the two researchers engaged in a systematic process. First, when coding discrepancies arose, both coders returned to the original interview transcripts to re-examine the relevant context. Second, they held collaborative discussions to articulate and compare their interpretations and coding rationale. Third, through iterative dialogue and critical examination of the data, consensus was achieved through negotiated agreement between the two researchers. The findings of three themes and six subthemes were checked and agreed upon by one nursing student who participated in the focus group, thus enhancing the realization of transferability to real-life scenarios. To ensure confirmability, notes were taken during the focus group interviews. Additionally, the research team regularly engaged in self-reflection by maintaining reflexive journals, in which researchers documented their assumptions, biases, and reactions throughout the data collection and analysis process. Making these subjective influences explicit and transparent, the research team was able to critically examine how their perspectives might shape data interpretation during team discussions. This process of ongoing self-reflection strengthened confirmability by ensuring that the findings were grounded in the data rather than influenced by researcher bias.

### Ethical considerations

This study received approval from the research ethics committee of a medical center. Informed consent was obtained from all participants, who were informed of their right to withdraw from the study without any reason. Each participant was offered a gift card worth $10 after the focus group interview.

## Results

Thirty-six nursing students (9 male and 27 female) participated in this study. The mean age of the participants was 20. 7 years old (Table 2). Three themes were identified: (1) educational entertainment (edutainment); (2) triggered motivation for enhanced learning; and (3) technological concerns and challenges (Table 3).

**Table 2:** Characteristics of nursing students (*N* = 36)

Variable	Mean ± SD/ *n* (%)
**Age**	20.7 ± 0.7
**Gender**	
Female	27 (75.0)
Male	9 (25.0)
**Experience of playing online games**	
No	2 (5.6)
Yes	34 (94.4)
**Experience of playing games (switch**,** X-Box**,** Wii)**	
No	4 (11.1)
Yes	32 (88.9)
**Experience of playing AR games**	
No	23 (63.9)
Yes	13 (36.1)
**Experience of playing VR games**	
No	15 (41.7)
Yes	21 (58.3)

AR: Augmented Reality; VR: virtual reality

**Table 3:** Subthemes and themes emerged

Subthemes	Themes
(1) Gamified learning (2) Novelty and enjoyment (1) Compete and eager to win (2) Concentrate and independent learning (1) Tension and discomfort (2) Unrealistic and casual attitude	Educational entertainment (Edutainment) Triggered motivation for enhanced learning Technological concerns and challenges

### Theme 1. Educational entertainment

Nursing students reported that using VR for intravenous injections training felt like playing an online game. They viewed VR as an effective edutainment tool. Many students appreciated VR learning because it provided a gamified experience, brought novelty to their education, and offered enjoyable learning experiences. Two subthemes emerged under this theme: (1) gamified learning, and (2) novelty and enjoyment.

#### Gamified learning

For nursing students, the process of intravenous injection VR feels like playing a game. They must complete each predefined step to progress to the next level, and a score is shown at the end. Many students enjoy this gamified learning approach, and some shared their experiences with completing the gamified learning of intravenous injection VR:

I feel the intravenous injection VR experience is exciting. It is different from being in a classroom. It is more like playing a game and does not feel rigid. I think this (VR) learning has better effects and makes it more enjoyable. (Participant No. 24)

I feel that the intravenous injection VR experience is quite similar to a game. In the end, you have a score presentation, just like a gaming experience. It makes me eager to pass the level. (Participant No. 33)

#### Novelty and enjoyment

Nursing students highlighted that the VR felt like a fun learning experience for their first experience of learning nursing techniques, which they found to be different from traditional learning methods. It was a novel and exciting experience for them. Some students shared their sense of novelty when entering the virtual environment for intravenous injections:

I find it quite novel because I had never tried this learning method before. I never thought that VR could be used in learning our professional subjects. I feel that VR can be integrated better into the context of intravenous injections because once I put on the headset, everything around me becomes a simulated hospital scenario. I like this design. (Participant No. 31)

When experiencing virtual reality. I found it interesting that there is a blood return phenomenon after the needle enters the vessel. I didn’t know how to handle that situation because I don’t have such experience in the training classroom. (Participant No. 4)

The design of the virtual patient’s hand is well done. One of the patient’s hands has an AV shunt, which vibrates if touched that hand. It helps you remember that you should not administer injections in that AV shunt hand in clinical practice. (Participant No. 32)

### Theme 2. Triggered motivation for enhanced learning

Nursing students were immersed in the VR environment independently, requiring them to focus on completing the task. The VR simulation provided a scoring system that assessed the correctness and completeness of their intravenous injection steps. This numerical feedback served multiple functions: it allowed students to self-assess their performance and informally compare results with peers. The visibility of scores and the opportunity for repeated practice created a competitive atmosphere that triggered students’ desire to achieve higher scores and outperform their previous attempts, thereby enhancing their learning motivation. Two subthemes emerged under this theme: (1) competition and the desire to win, and (2) concentrated and independent learning.

#### Competition and the desire to win

The design of the intravenous injection virtual reality in this study requires each step to be executed correctly to earn a score for that step. This score triggers a competitive mindset among nursing students as they strive to achieve high scores and compare themselves to their peers. This competition and the desire to excel psychologically stimulate nursing students’ learning motivation. Some students shared their experiences of competition in the intravenous injection virtual reality:

I think the score allows classmates to compare their scores and stimulates their desire to win. When I see other classmates making mistakes. I remind myself to pay attention to those steps. If my score is higher than theirs. I feel happy. (Participant No.10)

Another student shared that the presentation of scores helps them understand the correctness of their execution process and motivates them to try again for a higher score. The competitiveness they feel further fuels their enthusiasm for learning.

The final score quantifies the completion level of our technical skills. The results show where we went wrong, allowing us to improve. I think that’s great. It lets me know my ability to perform (intravenous injection) and increases my enthusiasm for learning. (Participant No.33)

#### Concentrated and independent learning

In traditional nursing skill practice, there would be teachers assisting or classmates available for discussion. However, in the VR environment, only the nursing student playing the nurse role interacts with the patient and performs intravenous injections. This gives nursing students a sense of being an independent nurse responsible for completing the intravenous injection. This concentrated and independent learning approach contributes to the students’ perceived effectiveness in their learning. Some nursing students shared their experiences:

In VR, the patients can talk to me, whereas in previous practices, they were manikins who didn’t provide any response. As a result, I might not be as meticulous in performing each step. With VR, I can focus even more on my learning. (Participant No. 27)

Traditional learning is like driving in a driving school where the instructor is like a coach observing how you drive, whereas VR experience is like driving on the road by yourself, making you more independent and focused. (Participant No. 10)

### Theme 3: Technological concerns and challenges

When nursing students use VR for intravenous injection learning, they experience a sense of nervousness due to the new learning method. They feel like they are playing a game and exhibit casual attitudes. Nursing students have shared concerns and challenges associated with VR use, including nervousness and discomfort, the perception of unrealistic elements, and a casual attitude toward the learning process. Two subthemes emerged under this theme: (1) tension and discomfort, and (2) unrealistic and casual attitude.

#### Tension and discomfort

Some nursing students experienced varying degrees of nervousness when faced with new VR equipment and entering the virtual patient room environment. They worry about operating the machine or talking with simulated patients, leading to different degrees of nervousness. Additionally, some students experience discomfort, such as dizziness or disorientation during the VR experience. Some nursing students shared their experiences:

This was my first experience with VR, and I felt nervous. Initially, I wondered if VR required much time to adapt to the hardware. However. after the teacher taught us how to operate it, we quickly became proficient. (Participant No.36)

I never thought VR technology could be combined with nursing. When I first put on the headset, I felt dizzy and my mind went blank. After wearing it for a while, I got used to it and entered a better state. (Participant No.34)

When playing VR, I already lost my sense of direction. When I took off the headset, I wondered—‘Why am I in this place? I was just in a faraway location.’ (Participant No.34)

#### Unrealistic and casual attitude

Nursing students felt a significant difference between operating the simulator joystick and the actual intravenous injections. This discrepancy made them perceive that the fine motor movements performed through the simulator joystick in VR were not realistic enough. Some students also expressed a casual attitude, considering it merely as a game. One student shared that the tactile sensations experienced during VR operation could not replace the sensory feedback of performing the actual procedure:

I feel that VR has a significant difference compared to previous learning. You don’t know what force to apply when performing the technique because a joystick controls it. So, you don’t know how much force to use when inserting the needle, even though the joystick is adjusted to the correct angle. It feels quite different to me. (Participant No.15)

Additionally, a small number of nursing students adopted a casual attitude during the gamified learning process.

The virtual reality experience of intravenous injections feels more like playing a game rather than performing a technique. It lacks the sense of accountability ‘Did I do something wrong? It doesn’t seem to matter.’ It allows for repeated attempts. However. compared to traditional methods of skill practice. VR provides a sense of overcoming challenges. (Participant No.33)

## Discussions

All the nursing students in this study were experiencing VR-based learning of nursing techniques for the first time. Three major themes emerged from the analysis of nursing students’ perceptions and experiences with VR: edutainment; triggered motivation for enhanced learning; and technological concerns and challenges. The findings highlight nursing students’ positive perceptions of their first VR-based learning experience, particularly in terms of how the edutainment features induced a sense of engagement and the immersive simulation triggered motivation that enhanced their learning; likewise, these findings also reveal the concerns experienced by students, such as technological challenges and initial physical discomfort with the VR equipment.

Nursing students perceived VR as an edutainment learning tool. This finding aligns with previous research [20, 21] regarding the novelty and engagement of VR learning. The impressive VR learning finding is also consistent with previous studies [22]. It demonstrates the appeal of VR as a learning modality for nursing students. By effectively utilizing VR for teaching, nursing educators can create a joyful and engaging learning environment for nursing students.

Among the nursing students in this study, 21 individuals (56.8%) had prior gaming experience with VR. The results showed that nursing students perceived the VR experience of intravenous injections as similar to playing a game. This finding is consistent with research on stroke patients undergoing rehabilitation therapy using VR or children’s VR experiences during the intravenous injections process, which also found the VR experience to be game-like and enjoyable [23, 24]. These results suggest that VR, whether used in healthcare or education, is an engaging therapeutic or learning tool that can provide users with enjoyment. Although this study indicated that VR made nursing students perceive the learning process as gamified and enjoyable, the research team specifically designed several impressive elements in the instructional design to enhance the learning outcomes.

This study focuses on nursing students learning intravenous injection techniques through VR gamified design. The thematic analysis revealed that VR stimulated students’ learning motivation for enhanced their learning outcomes, as evidenced by nursing students consistently described increased interest and improved understanding of IV injection procedures. These results align with previous studies that have found that the use of VR in teaching improve students’ learning motivation [6, 8, 25] and are consistent with earlier research demonstrating the positive effects of VR on student learning [17, 26]. However, a particular finding of this study indicates that the gamified nature of VR learning stimulates students’ learning motivation due to the competitive and achievement-oriented mindset fostered through game playing. Furthermore, this study found that nursing students perceived a significant difference between the virtual reality experience of intravenous injections and traditional technical practice. When experiencing VR, students were immersed alone in a virtual environment, allowing them to be more independent and focused on the task of intravenous injections, thereby enhancing their learning. Therefore, the findings of this qualitative study complement the limitations of some quantitative research results on VR and further explain why VR can enhance learning motivation and effectiveness. This is also the value of qualitative research for exploring the further cause reason.

The results of this study indicate that nursing students have concerns and challenges regarding using technology when experiencing VR for learning nursing techniques. These findings align with previous research identifying challenges and barriers perceived by nursing students [17, 27], highlighting the ongoing need to address issues when applying VR in nursing education. This study found that nursing students initially feel nervous when faced with and using new technological products. These findings are similar to previous research that identified a lack of familiarity with technologies [27]. Nursing students may experience anxiety when using new technologies but can quickly adapt after exposure [28]. These research findings demonstrate that students of this generation can quickly familiarize themselves with technological products. Therefore, we recommended that teachers effectively use new technologies or electronic products to engage students in their learning without worrying too much about the time required for students to learn the operating procedures. In this study, nursing students reported feeling nervousness when experiencing VR, indicating that they may feel slightly pressured when initially facing VR to learn nursing techniques. These findings do not entirely align with previous research that suggests VR provides a stress-free learning environment [17, 29, 30].

Some nursing students in this study reported feelings of dizziness and loss of orientation when initially wearing VR headsets. This finding is consistent with previous research that identified cybersickness and giddiness experienced by nursing students [2, 27, 31]. Therefore, to ensure student safety, instructors should brief students on VR experiences in advance and advise them to stop and rest immediately if they experience dizziness to prevent falls. Additionally, as students immerse themselves in a virtual environment during VR use, they may experience a loss of orientation in the real-world surroundings. Adequate spatial arrangements for VR experiences are essential to prevent students from colliding with objects.

The nursing students in this study perceived the VR experience as unrealistic. This finding is similar to previous research that found nursing students felt VR lacks a sense of reality [29] and experienced challenges and threats to actualization [21]. It shows that nursing students still feel it needs to be sufficiently real. Therefore, addressing the issue of realism is a challenge that VR currently needs to overcome. The nursing students in this study felt that the hand movements during injection were different from actual hand techniques, suggesting that enhancing the realism of hand movements is an area where VR design can be further improved or strengthened. Enhancing the realism of hand representations in VR simulators could significantly improve the fidelity of virtual reality training for healthcare professionals and manual clinical procedures.

There is currently limited qualitative research on nursing students’ VR learning experiences discussing their learning attitudes. However, this study found that a small number of nursing students developed a casual attitude due to the gamified nature of VR. This is something nursing instructors need to be aware of during class. Pre-class reminders are recommended for instructors to emphasize that although VR gamified learning is fun, students still need to approach virtual patient interactions with a genuine attitude and mood to truly learn effectively.

The findings of this study have important implications beyond intravenous injection training, suggesting potential transferability to other nursing skills and educational contexts. The three identified themes—edutainment, triggered motivation for enhanced learning, and technological concerns and challenges—are not unique to intravenous injections procedures but may apply broadly to other technical nursing competencies. For instance, the gamified learning approach and competitive elements that enhanced student engagement in this study could be similarly effective for VR-based training in catheterization, wound care, nasogastric tube insertion, or other invasive procedures. The benefits of concentrated and independent learning in a safe VR environment, which allowed students to practice without fear of harming patients, could be particularly valuable for high-risk procedures such as urinary catheterization or tracheostomy care. Furthermore, the immediate feedback and scoring mechanisms that triggered student motivation in this study could be incorporated into VR simulations for medication administration, patient assessment skills, or emergency response training. Future research could explore the applicability of these findings across a broader range of nursing procedures and investigate whether the identified themes remain consistent across different clinical skill domains.

### Study limitations

This study was conducted at a single nursing school in northern Taiwan, which may limit the generalizability of findings to other geographical locations, educational institutions, or cultural contexts. Potential power imbalances between instructors and students may have influenced participants’ responses, despite our efforts to ensure voluntary participation. To minimize this concern, interviews were conducted after course grades had been finalized. The nursing students in this study were using VR to learn nursing techniques for the first time. The VR device used was Oculus Rift S. Users may have different experiences using devices from different manufacturers and applying slightly different operational methods. Their usage frequency might also vary. Based on these findings, future research could track the experiences of VR learners across multiple learning sessions or involve the use of various nursing technologies to enhance knowledge in this field.

## Conclusion

This study demonstrates that VR is a viable instructional strategy for learning nursing techniques. Students perceived VR as an engaging educational tool that effectively integrates learning with enjoyment, fostering both competition and a sense of achievement through gamified experiences. These findings advance our understanding of VR’s role in enhancing student motivation and learning outcomes in nursing education.

## Data Availability

The datasets generated and/or analysed during the current study are not publicly available due to the data are in Chinese but are available from the corresponding author on reasonable request.
